# Phage protein Gp11 blocks *Staphylococcus aureus* cell division by inhibiting peptidoglycan biosynthesis

**DOI:** 10.1128/mbio.00679-24

**Published:** 2024-05-16

**Authors:** Qi Xu, Li Tang, Weilin Liu, Neng Xu, Yangbo Hu, Yong Zhang, Shiyun Chen

**Affiliations:** 1 CAS Key Laboratory of Special Pathogens and Biosafety, Wuhan Institute of Virology, Center for Biosafety Mega-Science, Chinese Academy of Sciences, Wuhan, China; 2 University of Chinese Academy of Sciences, Beijing, China; 3 State Key Laboratory of Virology, Wuhan Institute of Virology, Center for Biosafety Mega-Science, Chinese Academy of Sciences, Wuhan, China; The University of Tennessee Knoxville, Knoxville, Tennessee, USA

**Keywords:** phage, *Staphylococcus aureus*, essential gene, peptidoglycan, lipid II

## Abstract

Phages and bacteria have a long history of co-evolution. However, these dynamics of phage-host interactions are still largely unknown; identification of phage inhibitors that remodel host metabolism will provide valuable information for target development for antimicrobials. Here, we perform a comprehensive screen for early-gene products of ΦNM1 that inhibit cell growth in *Staphylococcus aureus*. A small membrane protein, Gp11, with inhibitory effects on *S. aureus* cell division was identified. A bacterial two-hybrid library containing 345 essential *S. aureus* genes was constructed to screen for targets of Gp11, and Gp11 was found to interact with MurG and DivIC. Defects in cell growth and division caused by Gp11 were dependent on MurG and DivIC, which was further confirmed using CRISPRi hypersensitivity assay. Gp11 interacts with MurG, the protein essential for cell wall formation, by inhibiting the production of lipid II to regulate peptidoglycan (PG) biosynthesis on the cell membrane. Gp11 also interacts with cell division protein DivIC, an essential part of the division machinery necessary for septal cell wall assembly, to disrupt the recruitment of division protein FtsW. Mutations in Gp11 result in loss of its ability to cause growth defects, whereas infection with phage in which the *gp11* gene has been deleted showed a significant increase in lipid II production in *S. aureus*. Together, our findings reveal that a phage early-gene product interacts with essential host proteins to disrupt PG biosynthesis and block *S. aureus* cell division, suggesting a potential pathway for the development of therapeutic approaches to treat pathogenic bacterial infections.

**IMPORTANCE:**

Understanding the interplay between phages and their hosts is important for the development of novel therapies against pathogenic bacteria. Although phages have been used to control methicillin-resistant *Staphylococcus aureus* infections, our knowledge related to the processes in the early stages of phage infection is still limited. Owing to the fact that most of the phage early proteins have been classified as hypothetical proteins with uncertain functions, we screened phage early-gene products that inhibit cell growth in *S. aureus*, and one protein, Gp11, selectively targets essential host genes to block the synthesis of the peptidoglycan component lipid II, ultimately leading to cell growth arrest in *S. aureus*. Our study provides a novel insight into the strategy by which Gp11 blocks essential host cellular metabolism to influence phage-host interaction. Importantly, dissecting the interactions between phages and host cells will contribute to the development of new and effective therapies to treat bacterial infections.

## INTRODUCTION

Phages are viruses that infect bacteria and are widely distributed in the biosphere (1). The “arms race” between phages and bacteria has contributed to rapid co-evolution and co-adaptation. Phages establish a successful infection and rapidly produce progeny viral particles that are a major force in reshaping host metabolism (2). Phage early proteins are toxic to or damage the host by immediately inhibiting host biosynthesis in the early stages of infection (3). Investigating the role of these putative phage proteins, particularly those that negatively affect host growth, will not only provide comprehensive insight into phage biology but will also help to better understand how bacteria are reprogrammed during phage infection. Several phage gene products that control host metabolism, e.g., host replication, transcription, and translation machinery, have been documented (4–6).

Phages have evolved strategies to hijack or manipulate their host’s biosynthetic pathways and machinery during infection, and therefore have evolved various mechanisms to control bacteria in long-term co-evolution and adaptation (7). The majority of known phage-host interactions occur early in the infection process (8), with phage early-gene products interacting with the host proteins to control and functionally shut them down. Studies have shown that phage proteins inhibit bacterial DNA biosynthesis by targeting host DNA polymerase and DNA gyrase (9, 10), and affect cell division by inhibiting the essential cell division proteins FtsZ and FtsL or the cytoskeletal protein MreB (11–14), or modifying or inhibiting bacterial RNA polymerase (15–18). In addition, other key host processes, such as quorum sensing and glycolysis, are also targeted by phages (19, 20).

Understanding the mechanisms by which phage proteins control the host will be valuable for the discovery of new antimicrobial drugs (5). In fact, the discovery of interactions between phage early proteins and essential bacterial proteins has been applied to high-throughput selection of antibacterial compounds (21). Moreover, the characterization of the active sites of the phage-host interaction will be useful for the development of effective approaches to identify small compounds or antibiotic peptides that mimic the antimicrobial activity of phage proteins (22, 23).

Although it is critical to understand the interaction between phages and bacteria, our knowledge of the different mechanisms that phages develop to control bacteria is still limited. Due to the lack of sequence similarity to known proteins and the lack of experimental evidence, most of the phage early proteins have been classified as hypothetical proteins with uncertain functions (24). Methicillin-resistant *Staphylococcus aureus* is one of the most common antibiotic-resistant bacterial pathogens and is considered as a major threat to human health, thus understanding phage-*S. aureus* interactions will help to develop phage-based antimicrobial strategies to control this notorious pathogen (21, 25).

In this study, we first comprehensively screened phage early proteins with growth control effects on *S. aureus*. One such early protein, Gp11, exacerbated division defects. We further demonstrated that Gp11 interacts with the peptidoglycan (PG) biosynthesis enzyme MurG and ultimately reduces the production of the final precursor molecule, lipid II. Gp11 also interacts with the cell division protein DivIC, which is essential for septal cell wall assembly by disrupting the recruitment of the division protein FtsW. Our results suggest that Gp11 interacts with essential host proteins to disrupt key PG biosynthesis and may provide a strategy to control *S. aureus* by targeting essential genes.

## RESULTS

### Gp11 causes growth defects and suppresses *S. aureus* cell division

Phage early proteins will “shut down” and “take over” the host, leading to host growth defects. To detect bacterial growth inhibition caused by hypothetical phage proteins, we performed a screening for 20 open reading frames (ORFs) of unknown function from *S. aureus* phage ΦNM1 (26). These genes were cloned into a plasmid driven by the isopropyl-β-D-1-thiogalactopyranoside (IPTG)-inducible promoter. The phage protein Gp104, which has been reported to inhibit the growth of *S. aureus* (21), was used as a positive control. After dot plating on a solid medium with IPTG induction, expression of *gp11* or *gp16* caused a reduction in the growth of *S. aureus* strain RN4220 (Fig. S1). Herein, we focused on characterizing the detailed roles of phage gene *gp11* as its overexpression showed the strongest inhibition of the growth of *S. aureus* (Fig. S1).

**Fig 1:**
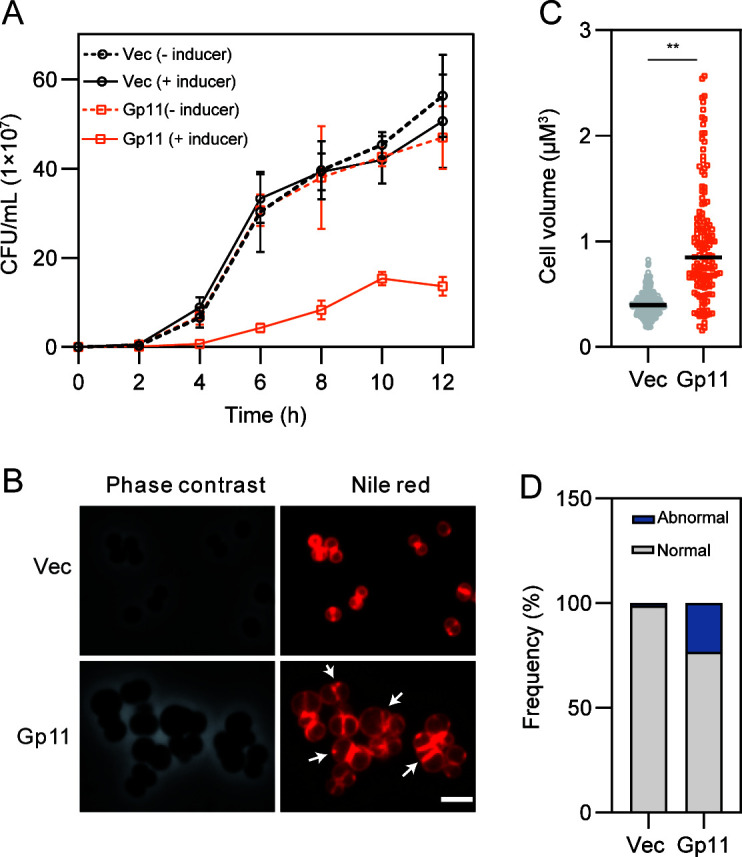
Overexpression of Gp11 in *S. aureus* alters cell morphology. (**A**) Effect of Gp11 overexpression on *S. aureus* cell growth. (**B**) Effect of Gp11 overexpression on *S. aureus* cell shape as visualized by phase-contrast and fluorescence microscopy. Arrows indicate septal defects as multiple. Cells were stained with membrane dye Nile Red. Scale bar, 2.5 µm. (**C**) Cell volume was measured using MicrobeJ. Cells were harvested after 2 h incubation with 10 µm NaAsO_2_. (**D**) Quantitation of cells with septal defects as multiple septa (abnormal), and nascent or complete septum (normal) after treatment with 10 µm NaAsO_2_ for 2 h. In this plot, *n* =  198 (Vec) and 91 (Gp11) cells; *P* values were determined by unpaired Student’s *t*-test. **P < 0.01 and *P < 0.05.

To exclude that the inhibitory effect caused by Gp11 was due to the IPTG induction system, a NaAsO_2_ induction system was introduced, and the same inhibitory effect was observed (Fig. 1A). Changes in cell morphology were observed after staining with the cell membrane fluorescent dye Nile Red (27). Compared to the control, overexpression of *gp11* resulted in a decrease in cell viability (Fig. 1A). Phenotypic defects of larger cell size and abnormal non-spherical morphology of cells were observed compared to the control (Fig. 1B). Strikingly, overexpression of *gp11* led to a fraction of cells with misplacement of nascent septa, and multiple parallel septa were frequently seen (Fig. 1B). Altered volume and abnormal cell division were also observed compared to the control. Cell volume increased significantly (Fig. 1C), and overexpression of *gp11* also led to the formation of abnormal cells with multiple septa (Fig. 1D). Taken together, these results suggest that Gp11 alters cell morphology and causes growth defects.

### Validation of MurG and DivIC as the targets of Gp11

**Fig 2:**
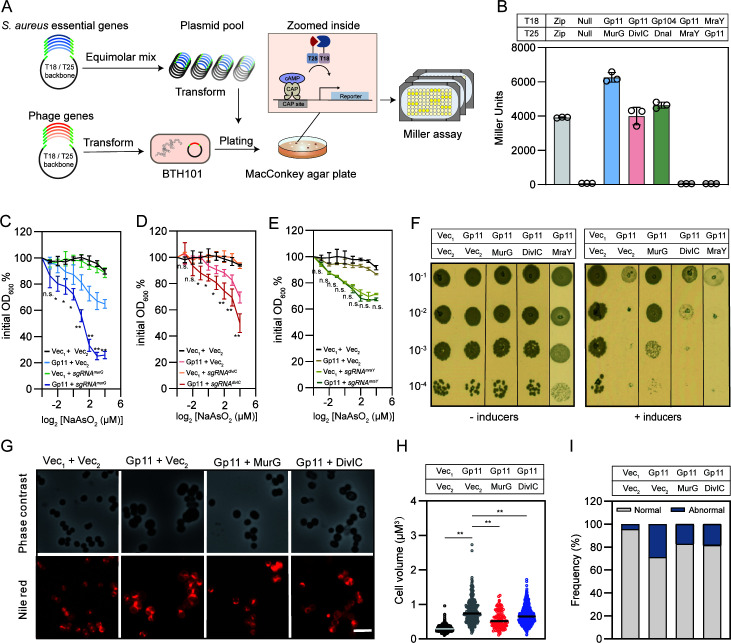
Validation of the targets of Gp11 in *S. aureus*. (**A**) Flowchart for screening Gp11-interacting proteins from *S. aureus* essential-gene BACTH libraries. With libraries being collected in an essential-gene plasmid pool and equimolar mix, the plasmid library was then transformed into *Escherichia coli* BTH101 reporter cells, which contain the phage gene, and plated on MacConkey agar plates. (**B**) In a bacterial two-hybrid assay of Gp11 interaction with MurG and DivIC, the known interaction pair Gp104 and DnaI was used as a positive control, while Gp11 and MraY as a negative control. (**C**) CRISPRi-based assay revealed MurG as the target of Gp11. The growth of *S. aureus* by knockdown of *murG* after expression of Gp11 (Gp11 + *sgRNA^murG^*) relative to no *sgRNA* control (Vec_1_ + Vec_2_). (**D**) The growth of *S. aureus* by knockdown of *divIC* after expression of Gp11 (Gp11 + *sgRNA^divIC^*) relative to no *sgRNA* control (Vec_1_ + Vec_2_). Each data point represents three independent replicates. *P* values were determined by unpaired Student’s *t*-test. **P < 0.01, *P < 0.05, and n.s.: no significant difference. (**E**) The growth of *S. aureus* by knockdown of *mraY* after expression of Gp11 (Gp11 + *sgRNA^mraY^*) relative to no sgRNA control (Vec_1_ + Vec_2_). (**F**) Growth assay of cells expressing Gp11, MurG, DivIC, or MraY on solid medium. (**G**) Microscopic images of *S. aureus* expressing Gp11, MurG, and DivIC. Cells were stained with membrane dye Nile Red. Scale bars, 2.5 µm. (**H**) Cell volume was measured using MicrobeJ. Cells were collected after 2 h incubation with 10 µM NaAsO_2_ and 100 µM IPTG. From left to right, *n* =  467, 418, 118, and 510 cells. *P* values were determined by unpaired Student’s *t*-test. **P < 0.01. (**I**) Quantitation of cells with septal defects (abnormal), and nascent or complete septum (normal) after cells were treated with 10 µM NaAsO_2_ and 100 µM IPTG for 2 h.

Having obtained the evidence that Gp11 causes growth defects, we hypothesized that it might interact with the essential genes in *S. aureus* by suppressing their functions. To identify such potential interacting genes, we constructed a bacterial adenylate cyclase-based two-hybrid (BACTH) essential gene library of *S. aureus*. Essential genes (*n* = 345) were cloned into plasmids pKT25 or pUT18, and Gp11 interaction targets were extensively screened (Fig. 2A). To test the feasibility of the screening approach with this library, we applied the known interaction pair Gp104 and DnaI as a positive control (28). As shown in Fig. 2B, Gp104 successfully interacts with DnaI, and Gp11 interacts with both MurG, which encodes a glycosyltransferase involved in PG biosynthesis, and DivIC, which is involved in cell division. A membrane protein called MraY, which is involved in PG synthesis but does not interact with Gp11, was chosen to test the specificity of this assay (Fig. 2B).

To further investigate if these interactions with Gp11 play a key role in *S. aureus*, we used CRISPRi hypersensitivity assay (29). By preventing transcription with a deactivated Cas9, CRISPRi allows sequence-specific knockdown of genes (Fig. S2A). Knockdown of *murG*, *divIC* significantly inhibited the growth of *S. aureus* (Fig. S2B); however, *murG* knockdown had no effect on bacterial morphology, whereas *divIC* knockdown showed significantly larger cell volumes (Fig. S2C). The phage-host interaction pair Gp104 and DnaI was used as a positive control, and Gp11 with its non-target DnaI was used as a negative control. The strain overexpressing Gp104 based on *dnaI* knockdown exhibited enhanced growth sensitivity compared to the control cells expressing Gp104 (Fig. S2D). However, reduction of *dnaI* levels by CRISPRi after expression of Gp11 showed no difference in growth (Fig. S2E). We conclude that the CRISPRi-based system effectively identifies phage-host interactions.

Next, we identified the targets of Gp11 in *S. aureus* using this CRISPRi hypersensitivity assay. Partial knockdown of the essential gene *murG* increased the sensitivity of cells overexpressing Gp11 (Fig. 2C). Furthermore, overexpression of Gp11 in the *divIC* knockdown cells sensitized the cells expressing Gp11 (Fig. 2D), further supporting our result that MurG and DivIC are physiologically important targets of Gp11 in *S. aureus*. Bacterial growth was impaired after *mraY* knockdown (Fig. S2B), but no difference was observed in Gp11-expressing cells with or without *mraY* knockdown (Fig. 2E).

Having confirmed that MurG and DivIC are the targets of Gp11, we hypothesized that growth inhibition by Gp11 could be rescued by overexpression of either MurG or DivIC. Moreover, as shown in Fig. 2F, cell growth could be partially restored when MurG or DivIC was co-expressed with Gp11 in *S. aureus*. In contrast to DivIC, MurG plays an important role in rescuing cell growth. As a negative control, MraY failed to rescue the growth inhibition caused by Gp11 (Fig. 2F). After staining the cells with the cell membrane fluorescent dye Nile Red, we used fluorescence microscopy to examine the cell morphological changes by evaluating the area of the cells. Cells co-expressing Gp11 with either MurG or DivIC restored the phenotypic defects (Fig. 2G) by showing a significant reversal in cell volume and reducing cell division defects compared to cells expressing Gp11 alone (Fig. 2H and I). Taken together, these results suggest that Gp11 controls cell growth by interacting with MurG and DivIC *in vivo*.

### Gp11 blocks cell division

**Fig 3:**
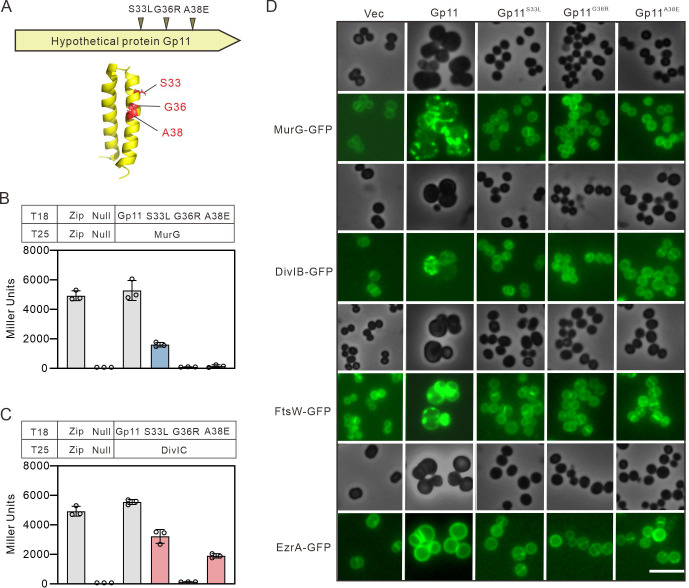
Gp11 acts to block cell division. (**A**) Summary of the identified mutants, all of which map to a hypothetical protein encoded by the gene *gp11* in *S. aureus* ΦNM1. The structural model of Gp11 predicted by AlphaFold (30), and the fraction of each mutant of Gp11 is indicated. (**B**) Analysis of the interactions between MurG and the mutants of Gp11 through the bacterial adenylate cyclase-based two-hybrid (BACTH) system. (**C**) Analysis of the interactions between DivIC and the mutants of Gp11 through BACTH. (**D**) The localization of MurG, DivIB, FtsW, and EzrA were monitored in the presence of Gp11 or its mutants. The cells were collected after 2 h incubation with 10 µM NaAsO_2_ and 100 µM IPTG. Scale bars, 2.5 µm.

Gp11 is a 53-amino-acid protein with a predicted double transmembrane helix (Fig. S3A). We examined Gp11 localization using Gp11-green fluorescent protein (GFP) fusion and found that Gp11 was indeed localized on the membrane (Fig. S3B). We performed a screen for Gp11-resistant mutants by generating transformants under conditions of high IPTG to identify key residues in Gp11. By sequencing of potential candidates, three ORF-resistant mutants were screened out as Gp11^S33L^, Gp11^G36R^, and Gp11^A38E^ (Fig. 3A; Fig. S3C). Expression of these point mutants revealed that all lost their ability to inhibit *S. aureus* growth (Fig. S3D and E), but remained localized on the membrane (Fig. S3B).

To further identify the key residues in Gp11 that interact with MurG and DivIC, we performed the BACTH assay. Mutation of G36 and A38 in Gp11 resulted in no interaction with MurG, whereas mutation of S33 decreased binding affinity to MurG (Fig. 3B). Regarding the interaction of Gp11 with DivIC, the A38E mutant does not make contact with MurG but still interacts with DivIC (Fig. 3C). These data indicate that Gp11 interacts with MurG and DivIC in a different pattern.

The division defects caused by Gp11 suggest that it disrupts the process of cell division. Using a MurG-GFP fusion protein, we observed that localization of MurG was frequently disrupted in cells expressing Gp11 (Fig. 3D). To confirm the role of the key residues of MurG in Gp11 function, the localization of MurG was assessed in the background of Gp11 expression or its point mutants. As shown in Fig. 3D, once the key amino acids were mutated, the localization of MurG was no longer affected. In addition, FtsW, the transglycosylase that is required for the formation of the septal cell wall, is recruited by the trimeric complex of DivIC, DivIB, and FtsL (31). We found that overexpression of Gp11, but not its mutants, disrupted the localization of FtsW, causing the fluorescence to be aggregated (Fig. 3D). We also observed fluorescence aggregation of DivIB, but the localization of the early division protein EzrA (32) appear to be unaffected in enlarged Gp11-expressing cells, cells with empty vector, or Gp11 mutants (Fig. 3D). These results suggest that Gp11 binds to MurG, resulting in its mis-localization, and interacts with DivIC to disrupt the recruitment of FtsW, which ultimately result in cell division defects.

### Gp11 spatially regulates PG biosynthesis in cell division and promotes phage infection

**Fig 4:**
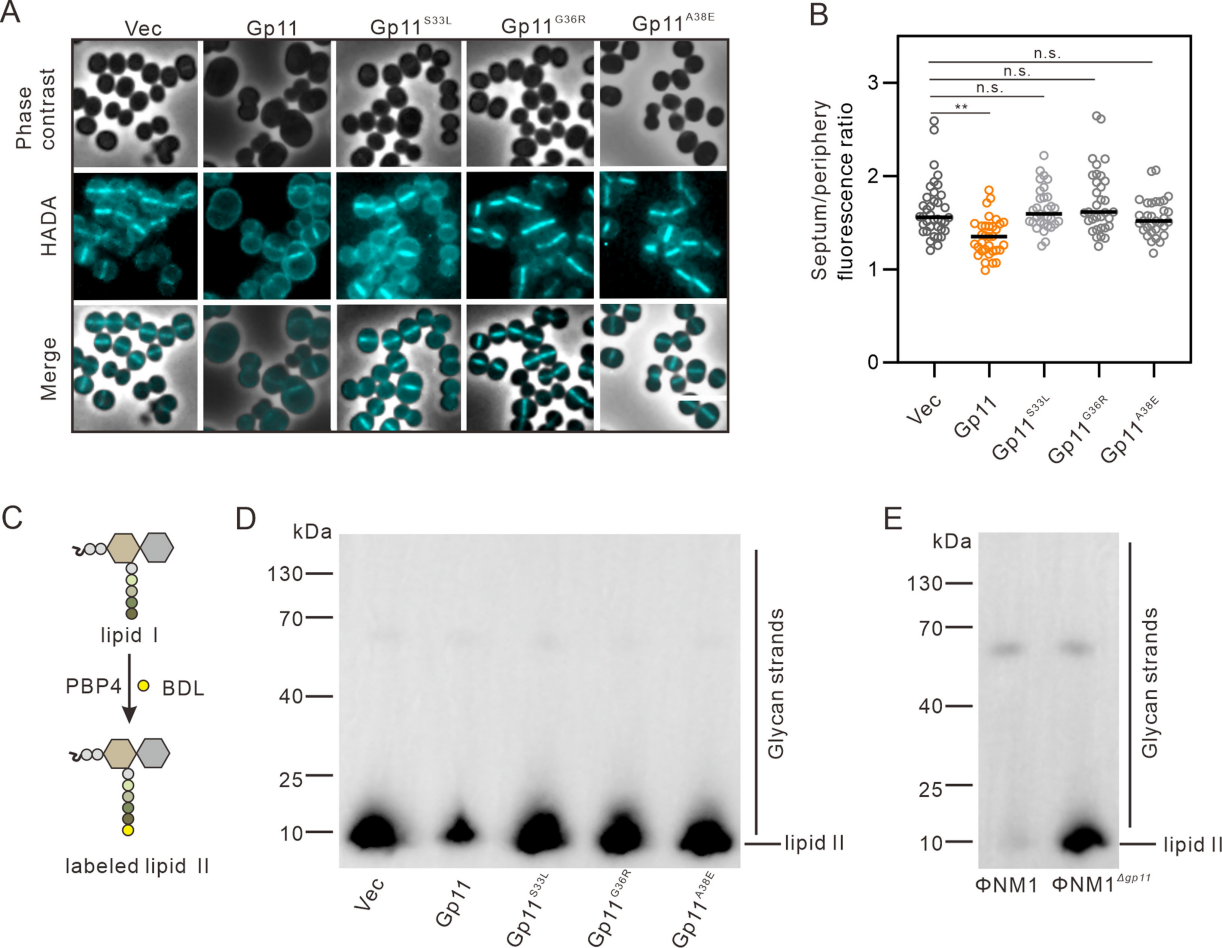
Gp11 targets peptidoglycan biosynthesis by inhibiting the activity of MurG. (**A**) *S. aureus* cells expressing Gp11 or its mutants were stained with the fluorescent cell wall marker D-amino acid 7-hydroxycoumarin carbonyl amino-D-alanine (HADA) and analyzed by phase-contrast and fluorescence microscopy. Scale bars, 2.5 µm. (**B**) Images of individual cells expressing Gp11 or its mutants were used to calculate the fluorescence ratio of the septal versus cell peripheral fluorescence signal. *n* ≥ 30. *P* values were determined by unpaired Student’s *t*-test. **P < 0.01 and n.s.: no significant difference. (**C**) The terminal D-Ala residue of the lipid II stem peptide was labeled with the biotin-D-lysine probe BDL using *S. aureus* PBP4 (34). (**D**) Western blot of lipid II after overexpression of Gp11 and its point mutants in *S. aureus* strain RN4220. (**E**) Western blot of lipid II after cells were infected with ΦNM1 and ΦNM1^Δ*gp11*^.

The interactions of Gp11 with MurG and DivIC led us to hypothesize that Gp11 may affect PG biosynthesis. To evaluate the effect of Gp11 on PG synthesis and the impact on *S. aureus* cell division, we labeled the cells with the fluorescent D-amino acid 7-hydroxycoumarin carbonyl amino-D-alanine (HADA), a molecule that is incorporated into newly synthesized PGs (33). Compared with the control cells, Gp11-overexpressing cells exhibited a more diffusible HADA signal, which was seen to occur at multiple sites within the cell, including peripheral and multiple partial septa (Fig. 4A), and the newly synthesized PG was depleted at the septum relative to the periphery in Gp11-overexpressing cells compared with controls (Fig. 4B), which is consistent with *murG* knockdown cells (Fig. S4A and B).

MurG is a glycosyltransferase in PG synthesis that transfers N-acetyl glucosamine (GlcNAc) from UDP-GlcNAc to the C4 position of lipid I to form a lipid-linked disaccharide peptide called lipid II (35). We therefore hypothesized that Gp11 targets MurG to inhibit its activity in producing lipid II, and performed an assay to detect cellular lipid II in *S. aureus* as previously described (34, 36). We isolated lipid II from Gp11-overexpressing cells, and the fraction was detected after labeling the stem peptides with biotin-D-lysine (BDL) for immunoblotting (Fig. 4C). As shown in Fig. 4D, a reduction in lipid II was observed in the presence of Gp11, but not its mutants, suggesting that Gp11 inhibits MurG activity, resulting in a reduction in lipid II. Taken together, our results indicate that Gp11 spatially regulates PG synthesis to control cell size.

**Fig 5:**
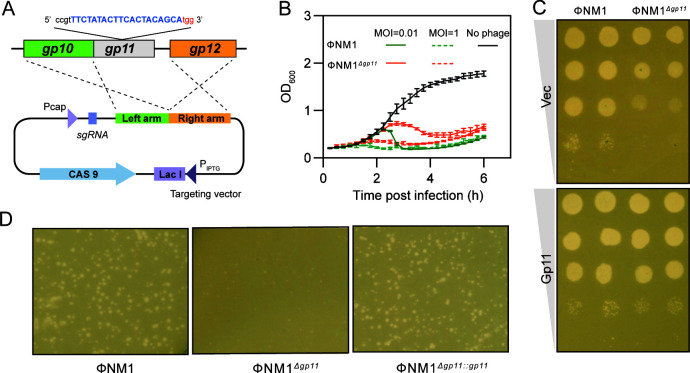
ΦNM1 *gp11* is contributed to phage infection. (**A**) The scheme for sgRNA-guided, CRISPR/Cas 9-induced homologous recombination in the *gp11* gene. (**B**) Growth curves of *S. aureus* RN4220 treated with ΦNM1 or ΦNM1*^Δgp11^* at a multiplicity of infection (MOI) of 0.01 or 1. (**C**) Serial, 10-fold dilutions of the phages ФNM1 or ΦNM1*^Δgp11^* spotted on lawns of cells harboring plasmid expressing Gp11 with 2.5 µM NaAsO_2_ or an empty vector (Vec). (**D**) Plaque morphologies of *S. aureus* RN4220 after infected with ФNM1, ФNM1*^Δgp11^* (delete the entire gene *gp11*), or its complementary phage ФNM1*^Δgp11::gp11^*. For complementation assay, ФNM1*^Δgp11^* was used to infect *S. aureus* RN4220, which contained the plasmid expressing *gp11* with 2.5 µM NaAsO_2_. The experiments were repeated at least three times.

Because Gp11 has been shown to affect lipid II production, we finally characterized its effect on PG biosynthesis during phage infection in the absence of *gp11*. We constructed an in-frame gene deletion of *gp11* named ΦNM1*^Δgp11^* and characterized the effect of Gp11 on phage fitness (Fig. 5A). ΦNM1*^Δgp11^* showed a reduced ability to lyse the host and resulted in smaller phage plaques compared to ΦNM1 (Fig. 5B and C). When Gp11 is expressed in the bacteria, the phage plaque size and phage infection are restored to the levels of wild type (Fig. 5C and D). This implies that Gp11 is an important phage protein involved in the takeover or manipulation of host bacteria.

Consistent with our finding that Gp11 overexpression reduced lipid II production, cells infected with ΦNM1*^Δgp11^* showed a significant increase in lipid II compared to the ΦNM1-infected strain 2 h after infection (Fig. 4E). Because MurG is a key enzyme in the PG synthesis pathway and directly regulates lipid II production, we therefore conclude that Gp11 inhibits MurG activity and blocks *S. aureus* cell division by inhibiting PG biosynthesis.

## DISCUSSION

With increasing concern about bacterial drug resistance, characterizing new targets has become a strong research focus in infection control. Phages engage in a long evolutionary arms race with their hosts over a period in which early phage proteins “take over” and “shut down” the host (37). Furthermore, bacterial proteins that are preferentially blocked by phage proteins have the potential to serve as novel antibacterial targets, as their disruption could disrupt key processes and slow bacterial growth. In the present study, we performed a comprehensive screening of phage early proteins that inhibit bacterial growth. We have identified a phage protein, Gp11, that inhibits cell division by interfering with PG synthesis. Our results suggest a model in which Gp11 interacts with MurG, leading to reduced production of lipid II, and interacts with DivIC to disrupt recruitment of the divisome complex.

We found that expression of *gp11* from phage ΦNM1 inhibits bacterial growth and alters cell morphology in *S. aureus* RN4220 with cell lysis (data not shown), which differs from observations of a lethal but not lytic effect of the phage protein E on *Staphylococcus carnosus* (38). This lysis effect is also observed in *Escherichia coli* with empty cell walls (39). The inhibitory effect of Gp11 could be used as a potentially beneficial strategy to combat *S. aureus*. Our results also suggest that Gp11 alters cell morphology by increasing host cell size. Phage adsorption and replication are closely related to the cell size of bacterial hosts, which affect the fitness and mutual evolution of phages and bacteria (40). The larger size caused by overexpression of Gp11 is consistent with the conclusion that Gp11 promotes phage infection (Fig. 5), supporting the idea that phages inhibit host cell division to propagate their progeny (14). These results suggest that Gp11 is a critical protein for phage ΦNM1 to control its host.

After screening for candidates that interact with phage proteins, we employed a CRISPRi hypersensitivity assay to validate the targets of phage proteins in *S. aureus*, which allows for the identification of direct phage targets. In this way, we found that Gp11 directly interacts with MurG and DivIC (Fig. 2B). These analyses provide a rapid method for identifying major targets of phages in the host using a dual screening model. As a glycosyltransferase, MurG is essential for bacterial survival by catalyzing the transfer of the GlcNAc motif of UDP-GlcNAc to the C4 hydroxyl MurNAc in lipid I to form lipid II (41) and is also an important target for the development of antimicrobial drugs. Elucidation of the mechanism by which Gp11 impairs cell growth requires a protein-protein interaction with MurG. This will provide new information for the development of novel drugs that directly target *S. aureus* in the future.

Gp11 inhibits cell growth by targeting PG biosynthesis in cell division. It has been reported that the correct localization of the division machinery in the midcell is correlated with PG density, which has been shown to be a marker of cell division (42). Gp11-mediated delocalization of MurG most likely disrupted its function spatiotemporally. Previous work has also shown that the phage protein DicB inhibits cell division by interacting with and affecting the localization of cell division proteins MinC and FtsZ (43, 44). Another phage protein Tip, which interacts with PilB, also antagonizes PilB function by delocalizing PilB from the poles (45). This strategy for phages to affect cell division by disrupting the localization of host division-associated proteins may be a general method for phages to alter host cell shape.

PG synthesis and cell division are inextricably linked, with the cell division septum being the main region of PG synthesis in *S. aureus* (46). Cell wall attack is one of the major strategies for phage to infect the host, but targeting PG synthesis is uncommon. A small protein like Gp11 targets the essential proteins MurG and DivIC to control its host. This implies the strong evolutionary pressure on phages to develop strategies to control host function by targeting two proteins simultaneously (19, 20, 47). DivIC is a key component of cell wall dynamics during division, which is essential for proper septum formation (48). The interaction between Gp11 and DivIC prevents the recruitment of FtsW, which interferes with PG biosynthesis at the septum, and overexpression of Gp11 showed a more diffuse HADA signal at the cell periphery (Fig. 4A). These data also support that loss of DivIC causes disruption of cell division as well as increases PG production at the cell periphery, which negatively impacts septum formation and ultimately results in thicker peripheral cell walls (49). During cell division, the late divisome proteins such as FtsW interact with other regulatory proteins to form the mature divisome and initiate septal PG synthesis (50). FtsW is a well-known lipid II-interacting protein (51). In a recent study, FtsW was reported to serve as a peptidoglycan polymerase that polymerizes lipid II at the septum into peptidoglycan (52). The reduction of lipid II in Gp11-expressing cells is consistent with the finding that FtsW was frequently mislocalized (Fig. 3D). We propose that mislocalization of FtsW may contribute to lipid II reduction during phage infection. Since the biosynthesis of the bacterial cell wall is closely coordinated with cell division (48, 53), we hypothesized that this could be a viable strategy for phages to influence PG synthesis during cell division and to lyse their host, which has been observed in *E. coli* (39, 54).

The target of Gp11 is relevant to its function as an inhibitor of PG synthesis. Gp11 inhibits the production of the final lipid-linked PG precursor, lipid II, by affecting the activity of MurG (Fig. 4). The altered morphological phenotype of the Gp11 mutant was accompanied by a reduction in lipid II, which is incorporated into the PG layer, helps to maintain cell shape, provides strength to withstand turgor, and is involved in the processes of cell growth and division (55). Notably, we found that infection with ФNM1*^Δgp11^* significantly increased the production of lipid II (Fig. 4E), which is consistent with previous studies that phage infection inhibits host PG biosynthesis. Previous work has shown that the phage protein Lys^M^ inhibits PG synthesis by targeting MurJ (56). The small RNA phage Qβ encoding the A_2_ protein blocked MurA, and the DNA phage ΦX174 encoding E protein inhibited MraY, both of which disrupt PG synthesis (54, 57). Although phage proteins have been shown to inhibit PG synthesis, the effect of this inhibition on the phage-infected cell has not been investigated. Here, we elucidated the effect of Gp11 on PG synthesis by comparing the results of wild-type ФNM1 and ФNM1*^Δgp11^* infections. Our results further explain that Gp11 inhibits *S. aureus* growth probably by affecting lipid II formation and thus preventing bacteria from maintaining normal morphology.

The exploitation of phage-host interaction has inspired the discovery of antibacterial targets (7). For example, a strategy to search for novel molecular targets of phages used time-resolved fluorescence resonance energy transfer to screen small molecules to disrupt the interaction between Gp104 and DnaI (21) and ultimately identified 11 molecules that were directly active against *S. aureus*. Recently, Zhang et al. (58) used small molecules to mimic the inhibitory effect of the phage protein Gp46 to develop a super-antibacterial drug that not only shortens the drug development cycle but also broadly inhibits most drug-resistant bacteria. Therefore, studying the Gp11-MurG interaction could provide information for the development of small molecules that mimic the inhibitory effect of Gp11. In summary, the strategy to affect cell division by disrupting the localization of host division-associated proteins may be a general way for phages to reshape the host, and the interaction partners identified here will lay the foundation for future antimicrobial treatment.

## MATERIALS AND METHODS

### Bacterial strains and growth conditions

The strains used in this study are listed in Table S1. Plasmids were cloned in *Escherichia coli* DH5α. The *E. coli* BL21(DE3) strain was used for protein overexpression and purification. *E. coli* strains were grown in Luria-Bertani (LB) broth or on LB agar supplemented with 100 µg mL^−1^ ampicillin or 50 µg mL^−1^ kanamycin. *S. aureus* strains were grown in tryptic soy broth (TSB; Difco) or on tryptic soy agar (TSA; Difco) supplemented with 10 µg mL^−1^ erythromycin or 10 µg mL^−1^ chloramphenicol when needed. To induce gene expression, 0.1 or 0.5 mM isopropyl-β-D-1-thiogalactopyranoside (IPTG) was added to the bacterial cultures.

### Plasmid construction

The plasmids used in this study are listed in Table S2. All oligonucleotides used in this study are listed in Table S3. Genes were cloned into plasmids using a ClonExpress II One-step Cloning Kit (Vazyme, Nanjing, China). Genomic DNA extracted from *S. aureus* strain RN4220 using a TIANAmp Bacteria DNA Kit (Tiangen, Beijing, China) was used as the template in the PCR reactions. The plasmid pTLS is for phage early protein overexpression in *S. aureus* and contains IPTG-inducible constructs with the Cm^R^ marker. All plasmids were constructed via multiple steps of restriction-ligation or Gibson assembly.

### Screening of phage genes that inhibit bacterial growth

To identify genes that inhibit bacterial growth, we cloned early genes of unknown function from ΦNM1 according to the previous RNA-seq results (59) into plasmid pTLS under the IPTG-inducible promoter. The plasmids were transformed into *S. aureus* RN4220 for a spot test. Cultures containing individual plasmids of the phage genes were inoculated onto TSA plates with or without 1 mM IPTG at 37°C for 12 h.

### Growth curves and spot dilution assay of *S. aureus*

For growth curve determination, overnight cultures of the strains were diluted 1:100 in TSB supplemented with 10 µM NaAsO_2_. Cells were grown at 37°C, and the optical density (OD)_600_ values were measured hourly using a plate reader (BioTek, Winooski, VT, USA). Cells used for dilutions and subsequent colony-forming unit (CFU) counting were taken from cultures under experimental conditions. Plates with dilutions that resulted in well-separated colonies were used for counting, and total CFUs were calculated. For the spot dilution assay, 10-fold serial dilutions of cultures were prepared in 500 µL phosphate-buffered saline (PBS), and 2 µL of these dilutions was plated on TSA and incubated at 37°C for 12 h.

### Construction and screening of the *S. aureus* essential gene BACTH library

A plasmid library containing 345 essential genes of *S. aureus* was constructed. The genes were amplified by PCR and ligated to the bacterial two-hybrid vector pUT18 and pKT25 provided in a BACTH System Kit (Euromedex, France). Plasmids were purified from the assembled clones to generate plasmid libraries. To screen the targets of phage proteins, 1 µL of the mixed essential gene plasmids was transformed into competent BTH101 cells harboring bait plasmids expressing phage proteins, and the transformants were plated on a MacConkey agar plate according to the manufacturer’s instructions. The red colonies were selected for assaying β-galactosidase activity (60).

### Bacterial two-hybrid assay

The BACTH system was used to detect protein-protein interactions (61). Briefly, the “bait” and “prey” proteins were fused to pKT25 and pUT18, respectively, and heat-shock–transformed into BTH101 competent cells lacking a functional *cyaA* gene. Transformants were grown on LB agar for 2 days at 30°C. Three clones were selected and grown for 16 h at 30°C with shaking in LB containing 50 µg mL^−1^ kanamycin, 100 µg mL^−1^ ampicillin, and 0.5 mM IPTG. The Miller assay was used to spectrophotometrically quantify β-galactosidase activity and to identify the protein-protein interactions (60). Results are representative of at least three independent replicates.

### CRISPRi hypersensitivity assay

CRISPRi-mediated inhibition of essential gene expression was performed as described (62). Using pISA-IPTG, the base-pairing region was designed to target the essential gene on the coding strand adjacent to the protospacer motif NGG. dCas9 protein expression was induced with IPTG. Results are representative of at least three independent replicates. To test the efficacy of the essential gene knockdown strain, relative growth in liquid TSB containing 1 mM IPTG was measured and compared to the growth of the no-sgRNA control at mid-log phase using a plate reader (BioTek). To verify the targets of Gp11 in *S. aureus*, a low inducible concentration was selected to knock down essential genes without affecting bacterial growth, and the plasmid pTR-*gp11* was transformed into a strain containing the CRISPRi system. The growth of CRISPRi *S. aureus* knockdown mutants relative to no-sgRNA control was measured after overexpression of Gp11 using a flat-bottomed 96-well plate.

### Labeling of *S. aureus* strains

The *S. aureus* strains were labeled with cell membrane fluorescent dye 9-diethylamino-5H-benzo [α] phenoxazine-5-one (Nile Red) as previously described for fluorescence microscopy (27). To label *S. aureus* membranes, cells were stained with Nile Red (MedChemExpress, New Jersey, USA) at a final concentration of 5 µg mL^−1^ for 5 min at room temperature, washed twice with PBS, and then 2 µL cultures was plated onto slides for imaging. To label the nascent PG of *S. aureus*, cells were incubated with fluorescent d-amino acid HADA (MedChemExpress) at a final concentration of 250 µM for 30 min at 37°C with shaking, washed with PBS to remove the unbound dye, and then plated on a slide for imaging (63, 64). The labeled cells were observed using phase contrast and the 4′,6-diamidino-2-phenylindole channel on an Olympus microscope.

### Microscopy and image analysis

To detect Gp11 overexpression in *S. aureus* growth, cells were stained with the cell membrane fluorescent dye Nile Red for 5 min at room temperature and washed twice with PBS before microscopy. To study the localization of Gp11 and its mutants, Gp11 or its mutants fused with GFP were cloned into expression vector pCI and then electrotransferred into *S. aureus*. Transformants were grown at 37°C in TSB medium, induced with 1 mM IPTG, and harvested by centrifugation at 5,000 × *g* for 3 min at OD_600_ = 0.4. Supernatants were decanted, and pellets were resuspended in PBS for imaging. To further evaluate the effect of Gp11 on MurG localization, Gp11 was co-transformed with MurG-GFP in *S. aureus*. Cells were harvested at OD_600_ between 0.4 and 0.6 with the addition of 10 µM NaAsO_2_ and 100 µM IPTG and then centrifuged at 5,000 × *g* for 3 min, the supernatant was discarded and washed twice with PBS and resuspended. Bacterial solution (2 µL) was placed on a slide for sampling and microscopic observation. The localization of the corresponding proteins was monitored by fluorescence visualization using an Olympus fluorescence microscope at 488 nm excitation. All images were taken on an Olympus inverted microscope at 10× and 100× magnification using a 100× oil immersion phase contrast objective. Microscope control and image acquisition were performed in NIS Elements (Nikon, Melville, NY, USA). Cell area was analyzed using the MicrobeJ (65) and ImageJ (66).

### Gp11 point mutant isolation

Point mutations of Gp11 were isolated as previously described (5, 13). Briefly, *S. aureus* RN4220 cells were transformed with pTLS-*gp11* and cultured overnight on TSB agar with 1 mM IPTG. The resistant colonies were harvested, and plasmids were extracted using a FastPure Plasmid Mini Kit (Vazyme). The plasmids were then retransformed into competent *S. aureus* RN4220 cells, and the resultant colonies were picked for spot dilution assay as described above to check growth inhibition. Plasmids contained in clones that no longer inhibited bacterial growth were subjected to sequencing analysis.

### Mutant construction of *gp11* in ΦNM1

The ΦNM1 *gp11* knockout strain was constructed using a CRISPR system. The plasmid pCas9 expressing Cas9 containing guide RNA spanning nucleotides 74–93 of *gp11* with the upstream and downstream fragments of *gp11* was transformed into *S. aureus* RN4220. A plaque assay was performed as previously described to select for the Gp11 knockout strain (26). Briefly, 100 µL *S*. *aureus* and 10 µL phage were mixed with 5 mL soft TSA and 5 mM CaCl_2_ and poured on TSA containing 1 mM IPTG and 10 µg mL^−1^ erythromycin. Plaques were selected for validation using the primers listed in Table S2.

### Phage infection assay

Overnight cultures of *S. aureus* RN4220 were diluted 1:100 in fresh TSB containing a final concentration of 5 mM CaCl_2_ to OD_600_ = 0.2, and 200  µL of cells was added to each well of a 96-well plate. ΦNM1 WT or ΦNM1*^Δgp11^* phages were immediately added to each well at the indicated multiplicity of infection of 1 and 0.01, respectively. After phage infection, growth was measured every 15 min at 37°C using a shaking plate reader (Biotek). The growth curve experiment was replicated at least three times independently.

### Lipid II purification

Lipid II extraction from *S. aureus* cells was performed as described (34). Briefly, overnight cultures of *S. aureus* WT or overexpressing Gp11 were diluted to OD_600_ = 0.01 and grown in TSB at 37°C to OD_600_ of 0.5–0.6. Cells were harvested after centrifugation at 4,000 × *g* for 10 min, and the pellets were resuspended in PBS and added to 8.75 mL CHCl_3_:MeOH (1:2). The mixture was vortexed at 25°C for 10 min and then centrifuged at 4,000 × *g* for 10 min to remove cell debris. The supernatant was transferred into a new centrifuge tube containing 5 mL CHCl_3_ and 3.75 mL PBS (pH 7.4). The mixture was vortexed for 10 min and centrifuged at 4,000 × *g* for 10 min to achieve phase separation. The material between the top aqueous and bottom organic layer was collected, dried in a vacuum desiccator, and resuspended in 20 µL of dimethyl sulfoxide (DMSO).

### Purification of *S. aureus* PBP4

The PBP4 protein was purified in *E. coli* BL21 (DE3) cells with a C-terminal His-tag as described previously (34). *E. coli* BL21 (DE3) cells carrying pET-28a-*pbp4* were grown in LB with 50 µg mL^–1^ kanamycin at 37°C. Overnight cultures were diluted 1:100 in fresh LB at 37°C to an OD_600_ of 0.4–0.6 and induced with 0.3 mM IPTG for 16 h at 25°C. Cells were collected by centrifugation for 10 min at 5,000 × *g*, and the pellet was resuspended in lysis buffer (20 mM Tris-HCl, pH 8, 150 mM NaCl, 10 mM imidazole, 10% glycerol). The cells were lysed by sonication and further centrifuged at 12,000 × *g* for 30 min. The supernatant was applied to a 5-mL HisTrap HP column (GE Healthcare, Chicago, IL, USA) equilibrated in lysis buffer. The column was extensively washed with wash buffer (20 mM Tris-HCl, pH 8, 150 mM NaCl, 30 mM imidazole, 10% glycerol), and proteins were eluted with elution buffer (20 mM Tris, pH 8.0, 150 mM NaCl, 10% glycerol, 250 mM imidazole).

### Western blot analysis of biotinylated lipid II

Biotinylation and detection of lipid II were performed as described (67). Briefly, 2 µL of lipid extraction solubilized in DMSO was added to a solution containing 4 µM PBP4, 3 mM BDL in reaction buffer (12.5 mM HEPES, 2 mM MnCl_2_, 250 µM Tween-80, pH 7.5) to a total volume of 10 µL. After incubation for 1.5 h at room temperature, the reaction was quenched with 10 µL of 2× SDS loading buffer. The final mixture (3 µL) was loaded onto a 4%–20% gradient polyacrylamide gel and run at 100 V for 1 h. The final mixture was transferred to an immunoblot polyvinylidene fluoride membrane (Bio-Rad, Hercules, CA, USA). BDL-lipid II was detected by blotting with streptavidin-horseradish peroxidase (1:10,000 dilution) (Beyotime, Shanghai, China).

### Statistical analysis

Statistical significance between two groups was analyzed by unpaired Student’s *t*-test (two-tailed) using GraphPad Prism 8 (GraphPad Software, Boston, MA, USA).

## SUPPLEMENTAL MATERIAL

Supplemental figures mbio.00679-24-s0001.docx Figures S1-S4.

Table S1 mbio.00679-24-s0002.xlsx Bacterial strains, phages, and plasmids used in this study.

Table S2 mbio.00679-24-s0003.xlsx Primers used in this study.

ASM does not own the copyrights to Supplemental Material that may be linked to, or accessed through, an article. The authors have granted ASM a non-exclusive, world-wide license to publish the Supplemental Material files. Please contact the corresponding author directly for reuse.

## References

[1] Dion MB, Oechslin F, Moineau S. 2020. Phage diversity, genomics and phylogeny. Nat Rev Microbiol 18:125–138. doi:10.1038/s41579-019-0311-5 32015529

[2] Wang YR, Fan HH, Tong YG. 2023. Unveil the secret of the bacteria and phage arms race. Int J Mol Sci 24:4363. doi:10.3390/ijms24054363 36901793 PMC10002423

[3] Roucourt B, Lavigne R. 2009. The role of interactions between phage and bacterial proteins within the infected cell: a diverse and puzzling interactome. Environ Microbiol 11:2789–2805. doi:10.1111/j.1462-2920.2009.02029.x 19691505

[4] Gopalkrishnan S, Ross W, Akbari MS, Li X, Haycocks JRJ, Grainger DC, Court DL, Gourse RL. 2022. Homologs of the Escherichia coli F element protein TraR, including phage lambda Orf73, directly reprogram host transcription. mBio 13:e0095222. doi:10.1128/mbio.00952-22 35583320 PMC9239242

[5] De Smet J, Wagemans J, Boon M, Ceyssens P-J, Voet M, Noben J-P, Andreeva J, Ghilarov D, Severinov K, Lavigne R. 2021. The bacteriophage LUZ24 "Igy" peptide inhibits the Pseudomonas DNA gyrase. Cell Rep 36:109567. doi:10.1016/j.celrep.2021.109567 34433028

[6] Mahata T, Molshanski-Mor S, Goren MG, Jana B, Kohen-Manor M, Yosef I, Avram O, Pupko T, Salomon D, Qimron U. 2021. A phage mechanism for selective nicking of dUMP-containing DNA. Proc Natl Acad Sci USA 118:e2026354118. doi:10.1073/pnas.2026354118 34074772 PMC8201957

[7] Wan X, Hendrix H, Skurnik M, Lavigne R. 2021. Phage-based target discovery and its exploitation towards novel antibacterial molecules. Curr Opin Biotechnol 68:1–7. doi:10.1016/j.copbio.2020.08.015 33007632

[8] Thammatinna K, Egan ME, Htoo HH, Khanna K, Sugie J, Nideffer JF, Villa E, Tassanakajon A, Pogliano J, Nonejuie P, Chaikeeratisak V. 2020. A novel vibriophage exhibits inhibitory activity against host protein synthesis machinery. Sci Rep 10:2347. doi:10.1038/s41598-020-59396-3 32047244 PMC7012835

[9] Kever L, Hünnefeld M, Brehm J, Heermann R, Frunzke J. 2021. Identification of Gip as a novel phage-encoded gyrase inhibitor protein of Corynebacterium glutamicum. Mol Microbiol 116:1268–1280. doi:10.1111/mmi.14813 34536319

[10] Son B, Patterson-West J, Arroyo-Mendoza M, Ramachandran R, Iben JR, Zhu J, Rao V, Dimitriadis EK, Hinton DM. 2021. A phage-encoded nucleoid associated protein compacts both host and phage DNA and derepresses H-NS silencing. Nucleic Acids Res 49:9229–9245. doi:10.1093/nar/gkab678 34365505 PMC8450097

[11] Mahata T, Molshanski-Mor S, Goren MG, Kohen-Manor M, Yosef I, Avram O, Salomon D, Qimron U. 2023. Inhibition of host cell division by T5 protein 008 (Hdi). Microbiol Spectr 11:e0169723. doi:10.1128/spectrum.01697-23 37888989 PMC10714956

[12] Bhambhani A, Iadicicco I, Lee J, Ahmed S, Belfatto M, Held D, Marconi A, Parks A, Stewart CR, Margolin W, Levin PA, Haeusser DP. 2020. Bacteriophage SP01 gene product 56 inhibits Bacillus subtilis cell division by interacting with FtsL and disrupting Pbp2B and FtsW recruitment. J Bacteriol 203:e00463-20. doi:10.1128/JB.00463-20 33077634 PMC7950406

[13] Molshanski-Mor S, Yosef I, Kiro R, Edgar R, Manor M, Gershovits M, Laserson M, Pupko T, Qimron U. 2014. Revealing bacterial targets of growth inhibitors encoded by bacteriophage T7. Proc Natl Acad Sci USA 111:18715–18720. doi:10.1073/pnas.1413271112 25512533 PMC4284602

[14] Kiro R, Molshanski-Mor S, Yosef I, Milam SL, Erickson HP, Qimron U. 2013. Gene product 0.4 increases bacteriophage T7 competitiveness by inhibiting host cell division. Proc Natl Acad Sci USA 110:19549–19554. doi:10.1073/pnas.1314096110 24218612 PMC3845191

[15] Zhao X, Chen C, Jiang X, Shen W, Huang G, Le S, Lu S, Zou L, Ni Q, Li M, Zhao Y, Wang J, Rao X, Hu F, Tan Y. 2016. Transcriptomic and metabolomic analysis revealed multifaceted effects of phage protein Gp70.1 on Pseudomonas aeruginosa. Front Microbiol 7:1519. doi:10.3389/fmicb.2016.01519 27725812 PMC5035744

[16] Nechaev S, Severinov K. 2003. Bacteriophage-induced modifications of host RNA polymerase. Annu Rev Microbiol 57:301–322. doi:10.1146/annurev.micro.57.030502.090942 14527281

[17] Gregory BD, Nickels BE, Garrity SJ, Severinova E, Minakhin L, Urbauer RJB, Urbauer JL, Heyduk T, Severinov K, Hochschild A. 2004. A regulator that inhibits transcription by targeting an intersubunit interaction of the RNA polymerase holoenzyme. Proc Natl Acad Sci USA 101:4554–4559. doi:10.1073/pnas.0400923101 15070756 PMC384785

[18] Minakhin L, Severinov K. 2005. Transcription regulation by bacteriophage T4 AsiA. Protein Expr Purif 41:1–8. doi:10.1016/j.pep.2004.09.019 15802215

[19] Zhang K, Li S, Wang Y, Wang Z, Mulvenna N, Yang H, Zhang P, Chen H, Li Y, Wang H, Gao Y, Wigneshweraraj S, Matthews S, Zhang K, Liu B. 2022. Bacteriophage protein PEIP is a potent Bacillus subtilis enolase inhibitor. Cell Rep 40:111026. doi:10.1016/j.celrep.2022.111026 35793626

[20] Shah M, Taylor VL, Bona D, Tsao Y, Stanley SY, Pimentel-Elardo SM, McCallum M, Bondy-Denomy J, Howell PL, Nodwell JR, Davidson AR, Moraes TF, Maxwell KL. 2021. A phage-encoded anti-activator inhibits quorum sensing in Pseudomonas aeruginosa. Mol Cell 81:571–583. doi:10.1016/j.molcel.2020.12.011 33412111

[21] Liu J, Dehbi M, Moeck G, Arhin F, Bauda P, Bergeron D, Callejo M, Ferretti V, Ha N, Kwan T, McCarty J, Srikumar R, Williams D, Wu JJ, Gros P, Pelletier J, DuBow M. 2004. Antimicrobial drug discovery through bacteriophage genomics. Nat Biotechnol 22:185–191. doi:10.1038/nbt932 14716317

[22] Chung IY, Kim BO, Han JH, Park J, Kang HK, Park Y, Cho YH. 2021. A phage protein-derived antipathogenic peptide that targets type IV pilus assembly. Virulence 12:1377–1387. doi:10.1080/21505594.2021.1926411 34008466 PMC8143254

[23] Ye F, Kotta-Loizou I, Jovanovic M, Liu X, Dryden DT, Buck M, Zhang X. 2020. Structural basis of transcription inhibition by the DNA mimic protein Ocr of bacteriophage T7. Elife 9:e52125. doi:10.7554/eLife.52125 32039758 PMC7064336

[24] Yin Y, Fischer D. 2008. Identification and investigation of ORFans in the viral world. BMC Genomics 9:24. doi:10.1186/1471-2164-9-24 18205946 PMC2245933

[25] Belley A, Callejo M, Arhin F, Dehbi M, Fadhil I, Liu J, McKay G, Srikumar R, Bauda P, Bergeron D, Ha N, Dubow M, Gros P, Pelletier J, Moeck G. 2006. Competition of bacteriophage polypeptides with native replicase proteins for binding to the DNA sliding clamp reveals a novel mechanism for DNA replication arrest in Staphylococcus aureus. Mol Microbiol 62:1132–1143. doi:10.1111/j.1365-2958.2006.05427.x 17010157

[26] Bae T, Baba T, Hiramatsu K, Schneewind O. 2006. Prophages of Staphylococcus aureus Newman and their contribution to virulence. Mol Microbiol 62:1035–1047. doi:10.1111/j.1365-2958.2006.05441.x 17078814

[27] Monteiro JM, Fernandes PB, Vaz F, Pereira AR, Tavares AC, Ferreira MT, Pereira PM, Veiga H, Kuru E, VanNieuwenhze MS, Brun YV, Filipe SR, Pinho MG. 2015. Cell shape dynamics during the staphylococcal cell cycle. Nat Commun 6:8055. doi:10.1038/ncomms9055 26278781 PMC4557339

[28] Hood IV, Berger JM. 2016. Viral hijacking of a replicative helicase loader and its implications for helicase loading control and phage replication. Elife 5:e14158. doi:10.7554/eLife.14158 27244442 PMC4887207

[29] Larson MH, Gilbert LA, Wang XW, Lim WA, Weissman JS, Qi LS. 2013. CRISPR interference (CRISPRi) for sequence-specific control of gene expression. Nat Protoc 8:2180–2196. doi:10.1038/nprot.2013.132 24136345 PMC3922765

[30] Jumper J, Evans R, Pritzel A, Green T, Figurnov M, Ronneberger O, Tunyasuvunakool K, Bates R, Žídek A, Potapenko A, et al.. 2021. Highly accurate protein structure prediction with AlphaFold. Nature 596:583–589. doi:10.1038/s41586-021-03819-2 34265844 PMC8371605

[31] Gamba P, Hamoen LW, Daniel RA. 2016. Cooperative recruitment of FtsW to the division site of Bacillus subtilis. Front Microbiol 7:1808. doi:10.3389/fmicb.2016.01808 27895631 PMC5108771

[32] Jorge AM, Hoiczyk E, Gomes JP, Pinho MG. 2011. EzrA contributes to the regulation of cell size in Staphylococcus aureus. PLoS One 6:e27542. doi:10.1371/journal.pone.0027542 22110668 PMC3215724

[33] Radkov AD, Hsu YP, Booher G, VanNieuwenhze MS. 2018. Imaging bacterial cell wall biosynthesis. Annu Rev Biochem 87:991–1014. doi:10.1146/annurev-biochem-062917-012921 29596002 PMC6287495

[34] Qiao Y, Srisuknimit V, Rubino F, Schaefer K, Ruiz N, Walker S, Kahne D. 2017. Lipid II overproduction allows direct assay of transpeptidase inhibition by beta-lactams. Nat Chem Biol 13:793–798. doi:10.1038/nchembio.2388 28553948 PMC5478438

[35] Men HB, Park P, Ge M, Walker S. 1998. Substrate synthesis and activity assay for MurG. J Am Chem Soc 120:2484–2485. doi:10.1021/ja974221p

[36] Qiao Y, Lebar MD, Schirner K, Schaefer K, Tsukamoto H, Kahne D, Walker S. 2014. Detection of lipid-linked peptidoglycan precursors by exploiting an unexpected transpeptidase reaction. J Am Chem Soc 136:14678–14681. doi:10.1021/ja508147s 25291014 PMC4210121

[37] Azam AH, Tanji Y. 2019. Bacteriophage-host arm race: an update on the mechanism of phage resistance in bacteria and revenge of the phage with the perspective for phage therapy. Appl Microbiol Biotechnol 103:2121–2131. doi:10.1007/s00253-019-09629-x 30680434

[38] Halfmann G, Götz F, Lubitz W. 1993. Expression of bacteriophage PhiX174 lysis gene E in Staphylococcus carnosus TM300. FEMS Microbiol Lett 108:139–143. doi:10.1111/j.1574-6968.1993.tb06089.x 8486239

[39] Witte A, Wanner G, Sulzner M, Lubitz W. 1992. Dynamics of Phix174 protein E-mediated lysis of Escherichia-coli. Arch Microbiol 157:381–388. doi:10.1007/BF00248685 1534215

[40] Choua M, Heath MR, Bonachela JA. 2021. Evolutionarily stable coevolution between a plastic lytic virus and its microbial host. Front Microbiol 12:637490. doi:10.3389/fmicb.2021.637490 34093461 PMC8172972

[41] Mohammadi T, Karczmarek A, Crouvoisier M, Bouhss A, Mengin-Lecreulx D, den Blaauwen T. 2007. The essential peptidoglycan glycosyltransferase MurG forms a complex with proteins involved in lateral envelope growth as well as with proteins involved in cell division in Escherichia coli. Mol Microbiol 65:1106–1121. doi:10.1111/j.1365-2958.2007.05851.x 17640276 PMC2170320

[42] Do T, Schaefer K, Santiago AG, Coe KA, Fernandes PB, Kahne D, Pinho MG, Walker S. 2020. Staphylococcus aureus cell growth and division are regulated by an amidase that trims peptides from uncrosslinked peptidoglycan. Nat Microbiol 5:291–303. doi:10.1038/s41564-019-0632-1 31932712 PMC7046134

[43] Johnson JE, Lackner LL, Hale CA, de Boer PAJ. 2004. ZipA is required for targeting of (D)MinC/DicB, but not (D)MinC/MinD, complexes to septal ring assemblies in Escherichia coli. J Bacteriol 186:2418–2429. doi:10.1128/JB.186.8.2418-2429.2004 15060045 PMC412171

[44] Johnson JE, Lackner LL, de Boer PAJ. 2002. Targeting of (D)MinC/MinD and (D)MinC/DicB complexes to septal rings in Escherichia coli suggests a multistep mechanism for MinC-mediated destruction of nascent FtsZ rings. J Bacteriol 184:2951–2962. doi:10.1128/JB.184.11.2951-2962.2002 12003935 PMC135045

[45] Chung IY, Jang HJ, Bae HW, Cho YH. 2014. A phage protein that inhibits the bacterial ATPase required for type IV pilus assembly. Proc Natl Acad Sci USA 111:11503–11508. doi:10.1073/pnas.1403537111 25049409 PMC4128137

[46] Wacnik K, Rao VA, Chen X, Lafage L, Pazos M, Booth S, Vollmer W, Hobbs JK, Lewis RJ, Foster SJ. 2022. Penicillin-binding protein 1 (PBP1) of Staphylococcus aureus has multiple essential functions in cell division. mBio 13:e0066922. doi:10.1128/mbio.00669-22 35703435 PMC9426605

[47] Brüssow H, Hendrix RW. 2002. Phage genomics: small is beautiful. Cell 108:13–16. doi:10.1016/s0092-8674(01)00637-7 11792317

[48] Monteiro JM, Pereira AR, Reichmann NT, Saraiva BM, Fernandes PB, Veiga H, Tavares AC, Santos M, Ferreira MT, Macário V, VanNieuwenhze MS, Filipe SR, Pinho MG. 2018. Peptidoglycan synthesis drives an FtsZ-treadmilling-independent step of cytokinesis. Nature 554:528–532. doi:10.1038/nature25506 29443967 PMC5823765

[49] Tinajero-Trejo M, Carnell O, Kabli AF, Pasquina-Lemonche L, Lafage L, Han A, Hobbs JK, Foster SJ. 2022. The Staphylococcus aureus cell division protein, DivIC, interacts with the cell wall and controls its biosynthesis. Commun Biol 5:1228. doi:10.1038/s42003-022-04161-7 36369270 PMC9652317

[50] den Blaauwen T, Hamoen LW, Levin PA. 2017. The divisome at 25: the road ahead. Curr Opin Microbiol 36:85–94. doi:10.1016/j.mib.2017.01.007 28254403 PMC6436919

[51] Welsh MA, Schaefer K, Taguchi A, Kahne D, Walker S. 2019. Direction of chain growth and substrate preferences of shape, elongation, division, and sporulation-family peptidoglycan glycosyltransferases. J Am Chem Soc 141:12994–12997. doi:10.1021/jacs.9b06358 31386359 PMC6738341

[52] Taguchi A, Welsh MA, Marmont LS, Lee W, Sjodt M, Kruse AC, Kahne D, Bernhardt TG, Walker S. 2019. FtsW is a peptidoglycan polymerase that is functional only in complex with its cognate penicillin-binding protein. Nat Microbiol 4:587–594. doi:10.1038/s41564-018-0345-x 30692671 PMC6430707

[53] Puls JS, Brajtenbach D, Schneider T, Kubitscheck U, Grein F. 2023. Inhibition of peptidoglycan synthesis is sufficient for total arrest of staphylococcal cell division. Sci Adv 9:eade9023. doi:10.1126/sciadv.ade9023 36947615 PMC10032595

[54] Orta AK, Riera N, Li YE, Tanaka S, Yun HG, Klaic L, Clemons WM. 2023. The mechanism of the phage-encoded protein antibiotic from PhiX174. Science 381:eadg9091. doi:10.1126/science.adg9091 37440661 PMC12747129

[55] Vollmer W, Blanot D, de Pedro MA. 2008. Peptidoglycan structure and architecture. FEMS Microbiol Rev 32:149–167. doi:10.1111/j.1574-6976.2007.00094.x 18194336

[56] Chamakura KR, Sham LT, Davis RM, Min L, Cho H, Ruiz N, Bernhardt TG, Young R. 2017. A viral protein antibiotic inhibits lipid II flippase activity. Nat Microbiol 2:1480–1484. doi:10.1038/s41564-017-0023-4 28894177 PMC5764540

[57] Bernhardt TG, Wang IN, Struck DK, Young R. 2001. A protein antibiotic in the phage Q beta virion: diversity in lysis targets. Science 292:2326–2329. doi:10.1126/science.1058289 11423662

[58] Zhang P, Zhao X, Wang Y, Du K, Wang Z, Yu J, Chang G, Matthews S, Wang H, Liu B. 2022. Bacteriophage protein Gp46 is a cross-species inhibitor of nucleoid-associated HU proteins. Proc Natl Acad Sci USA 119:e2116278119. doi:10.1073/pnas.2116278119 35193978 PMC8892312

[59] Goldberg GW, Jiang W, Bikard D, Marraffini LA. 2014. Conditional tolerance of temperate phages via transcription-dependent CRISPR-Cas targeting. Nature 514:633–637. doi:10.1038/nature13637 25174707 PMC4214910

[60] Hu Y, Wang Z, Feng L, Chen Z, Mao C, Zhu Y, Chen S. 2016. sigma(E) -dependent activation of RbpA controls transcription of the furA-katG operon in response to oxidative stress in mycobacteria. Mol Microbiol 102:107–120. doi:10.1111/mmi.13449 27353316

[61] Karimova G, Pidoux J, Ullmann A, Ladant D. 1998. A bacterial two-hybrid system based on a reconstituted signal transduction pathway. Proc Natl Acad Sci USA 95:5752–5756. doi:10.1073/pnas.95.10.5752 9576956 PMC20451

[62] Peters JM, Colavin A, Shi H, Czarny TL, Larson MH, Wong S, Hawkins JS, Lu CHS, Koo BM, Marta E, Shiver AL, Whitehead EH, Weissman JS, Brown ED, Qi LS, Huang KC, Gross CA. 2016. A comprehensive, CRISPR-based functional analysis of essential genes in bacteria. Cell 165:1493–1506. doi:10.1016/j.cell.2016.05.003 27238023 PMC4894308

[63] Peters K, Pazos M, VanNieuwenhze MS, Vollmer W. 2019. Optimized protocol for the incorporation of FDAA (HADA labeling) for in situ labeling of peptidoglycan. Bio Protoc 9:e3316. doi:10.21769/BioProtoc.3316 PMC7854060 33654824

[64] Kuru E, Tekkam S, Hall E, Brun YV, Van Nieuwenhze MS. 2015. Synthesis of fluorescent D-amino acids and their use for probing peptidoglycan synthesis and bacterial growth in situ. Nat Protoc 10:33–52. doi:10.1038/nprot.2014.197 25474031 PMC4300143

[65] Ducret A, Quardokus EM, Brun YV. 2016. MicrobeJ, a tool for high throughput bacterial cell detection and quantitative analysis. Nat Microbiol 1:16077. doi:10.1038/nmicrobiol.2016.77 27572972 PMC5010025

[66] Schneider CA, Rasband WS, Eliceiri KW. 2012. NIH Image to ImageJ: 25 years of image analysis. Nat Methods 9:671–675. doi:10.1038/nmeth.2089 22930834 PMC5554542

[67] Lebar MD, Lupoli TJ, Tsukamoto H, May JM, Walker S, Kahne D. 2013. Forming cross-linked peptidoglycan from synthetic gram-negative Lipid II. J Am Chem Soc 135:4632–4635. doi:10.1021/ja312510m 23480167 PMC3658469

