# Supplementary material for: Phage protein Gp11 blocks Staphylococcus aureus cell division by inhibiting peptidoglycan biosynthesis
Source: mBio. 2024 May 16;15(6):e00679-24. doi: 10.1128/mbio.00679-24 (PMC11237401; doi:10.1128/mbio.00679-24)
Supplement: Supplemental figures — Figures S1-S4. [file mbio.00679-24-s0001.docx]

**Supporting information**

**Supplementary Figures**


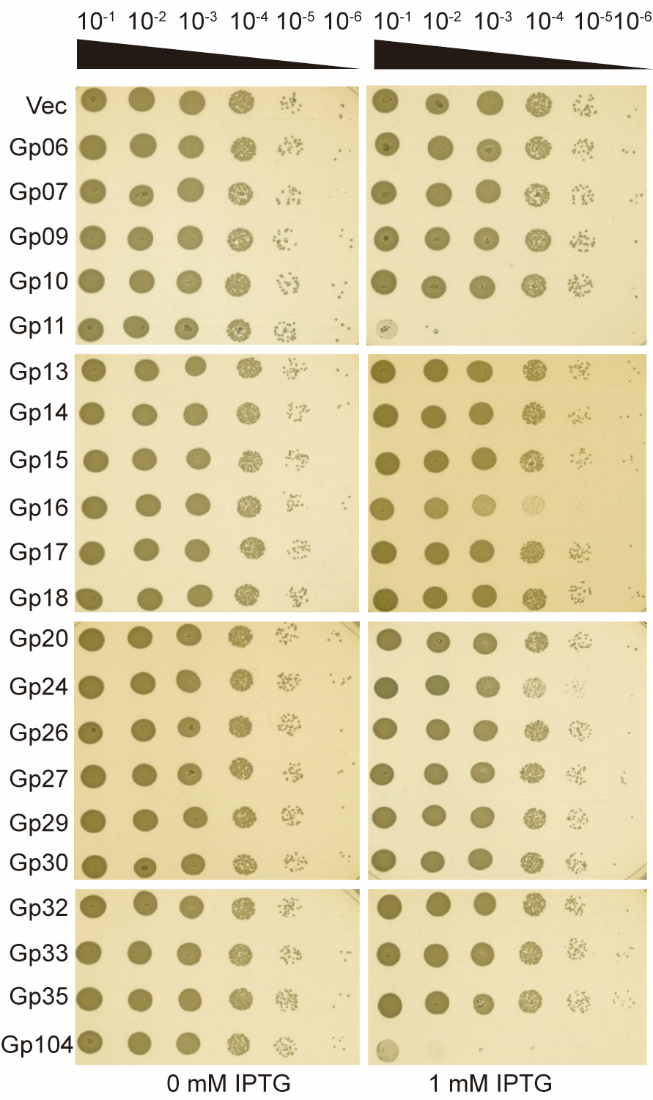


**Figure S1. Screening of phage early gene products that inhibit *S. aureus* growth**

Serial dilutions of *S. aureus* cells expressing phage early gene products from an IPTG-inducible promoter on TSA plates without (left) or with 1 mM IPTG (right). The empty vector was used as a negative control and Gp104 was used as a positive control.


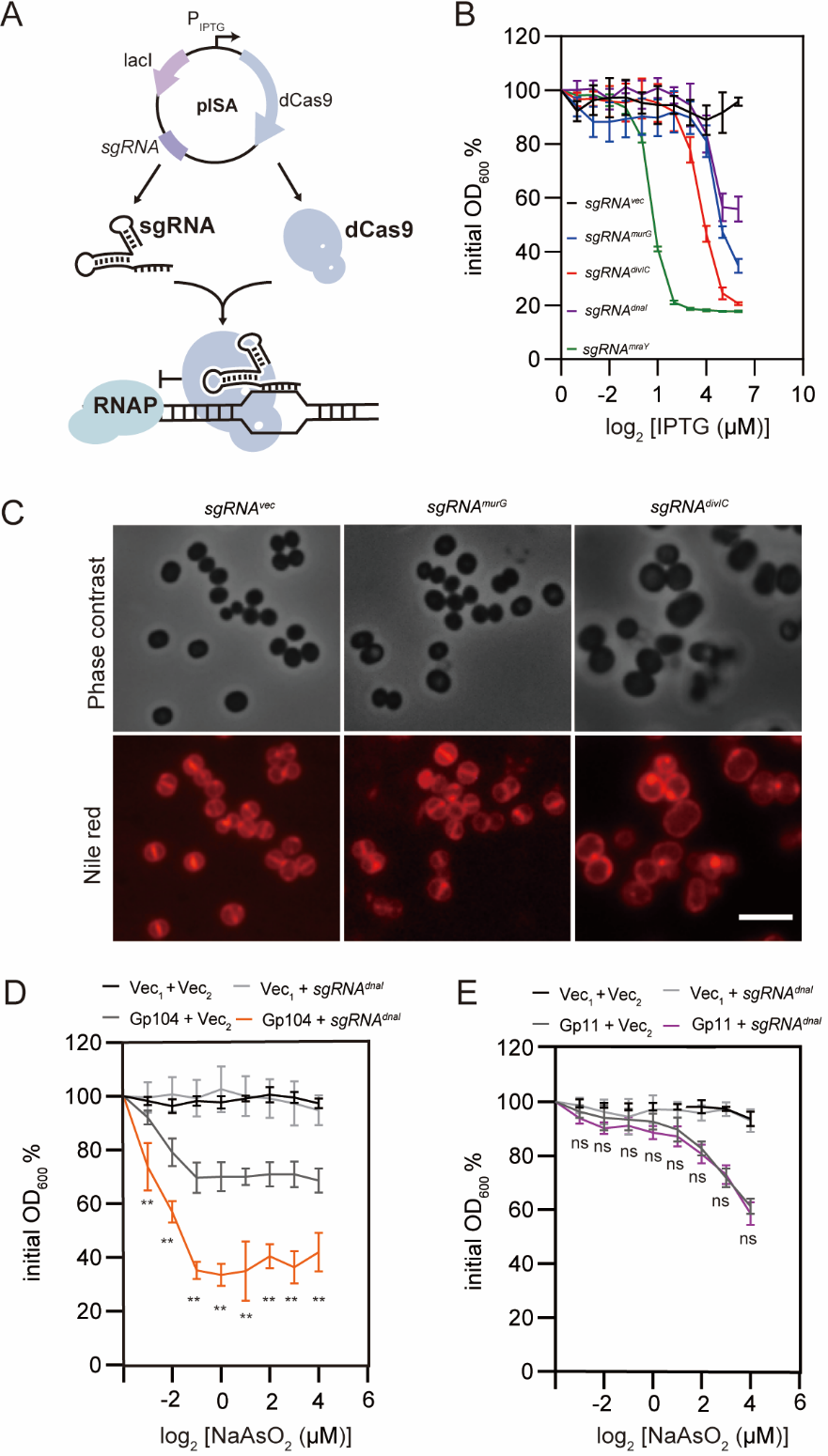


**Figure S2. Construction and test of a CRISPRi system in *S. aureus***

(A) *S. aureus* IPTG-inducible dCas9 binds to gene targets by specifically expressed sgRNAs to prevent the progression of RNA polymerase (RNAP), which suppresses gene transcription. (B) Effect of CRISPRi-mediated inhibition of essential gene expressions. (C) Micrographs of Nile Red labeled *S. aureus* RN4220 cells by knocking down of the *murG* or *divIC* gene. Scale bar, 2.5 µm. (D and E) Growth curves of *S. aureus* cells by knocking down of *dnaI* relative to no sgRNA control after expressing of Gp104 (D) or Gp11 (E). **p < 0.01, *p < 0.05. n.s. no significant difference by unpaired Student’s *t*-test.


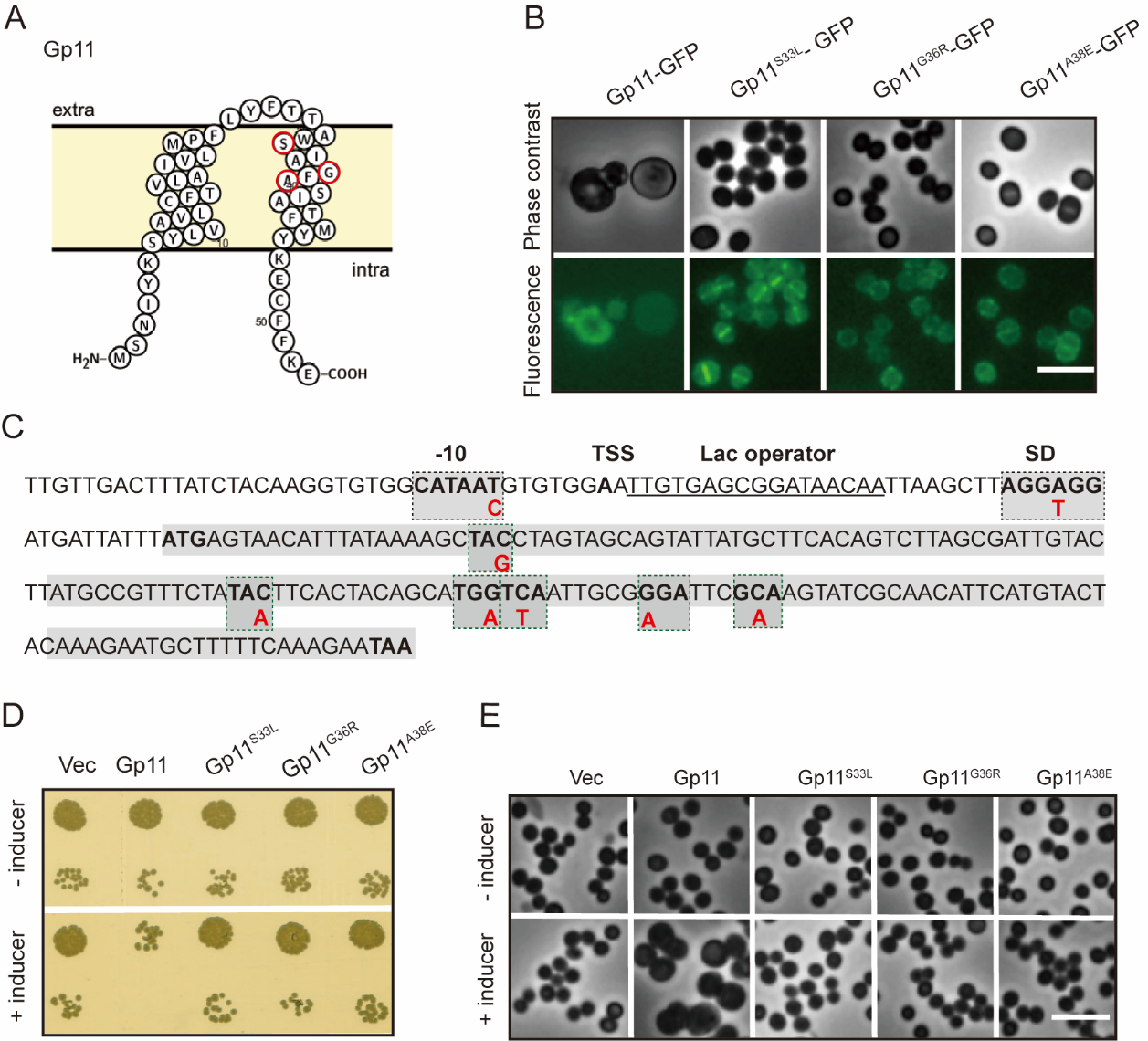


**Figure S3. The effect of Gp11 and its point mutants on *S. aureus* cell morphology**

(A) A predicted topological model of Gp11 by using Protter. The three mutated amino acids of Gp11 are colored with red circles. (B) Microscopy images of cells overexpressing Gp11 or its three point mutants C-terminally tagged with GFP. The GFP signal shows fusion protein localization. Scale bar, 2.5 µm. (C) The coding sequence of *gp11* and the inducing promoter. Mutated nucleotides are indicated by red color. (D) Growth inhibitory effect of *S. aureus* cells overexpressing of Gp11 or its three point mutants on the TSA plates without (above) or with (bottom) 10 µM NaAsO_2_. (E) Overexpressing of Gp11 or its three point mutants on cell morphology. Scale bar, 2.5 µm.


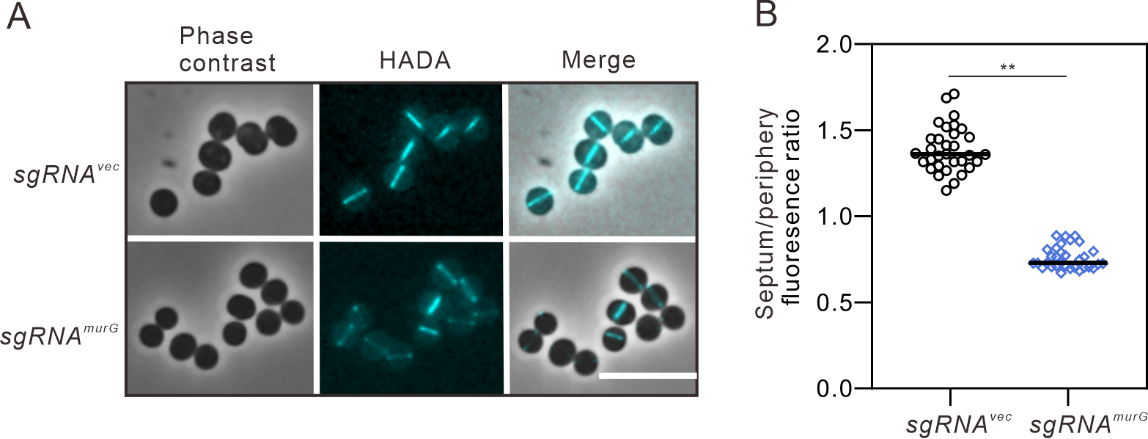


**Figure S4. MurG regulates *S. aureus* PG synthesis**

(A) Images of *murG* knockdown (*sgRNA^murG^*) and its control (*sgRNA^vec^*) cells incubated with HADA to follow PG synthesis observed under phase-contrast and fluorescence microscope. Scale bars, 2.5 µm. (B) The fluorescence ratio of septum versus periphery in *S. aureus* knockdown (*sgRNA^murG^*) and its control (*sgRNA^vec^*) cells (from A). n ≥ 30. P-values were determined by unpaired Student’s *t*-test. **p < 0.01; n.s. no significant difference.
